# Molecular Recognition Effects in Atomistic Models of Imprinted Polymers

**DOI:** 10.3390/ijms12084781

**Published:** 2011-07-28

**Authors:** Eduardo M. A. Dourado, Carmelo Herdes, Paul R. van Tassel, Lev Sarkisov

**Affiliations:** 1 Institute for Materials and Processes, School of Engineering, University of Edinburgh, Edinburgh, Midlothian EH9 3JL, UK; E-Mail: e.dourado@ed.ac.uk; 2 Centro de Química de Évora, Universidade de Évora, Rua Romão Romalho 59, 7000 Évora, Portugal; E-Mail: cherdes@uevora.pt; 3 Department of Chemical and Environmental Engineering, Yale University, New Haven, CT 06520-8286, USA; E-Mail: paul.vantassel@yale.edu

**Keywords:** molecular recognition, imprinted polymer, simulation, adsorption, rebinding, Monte Carlo, dynamics

## Abstract

In this article we present a model for molecularly imprinted polymers, which considers both complexation processes in the pre-polymerization mixture and adsorption in the imprinted structures within a single consistent framework. As a case study we investigate MAA/EGDMA polymers imprinted with pyrazine and pyrimidine. A polymer imprinted with pyrazine shows substantial selectivity towards pyrazine over pyrimidine, thus exhibiting molecular recognition, whereas the pyrimidine imprinted structure shows no preferential adsorption of the template. Binding sites responsible for the molecular recognition of pyrazine involve one MAA molecule and one EGDMA molecule, forming associations with the two functional groups of the pyrazine molecule. Presence of these specific sites in the pyrazine imprinted system and lack of the analogous sites in the pyrimidine imprinted system is directly linked to the complexation processes in the pre-polymerization solution. These processes are quite different for pyrazine and pyrimidine as a result of both enthalpic and entropic effects.

## 1. Introduction

A recent, excellent review by Nicholls and co-workers highlights an important and growing role of computer simulations and theoretical approaches in the field of molecularly imprinted polymers (MIPs) [1]. Common stages of a MIP’s lifecycle include preparation of a pre-polymerization mixture, initiation and polymerization, template and solvent removal and, finally, the actual function of the material as an adsorbent, chromatographic stationary phase or in some other capacity. Computer simulations can provide important fundamental insights into the molecular details of the processes associated with all these stages, thus guiding the design and optimization of new materials. Over the years, however, these various stages have received rather unequal attention in the literature.

The extent of complexation between the functional monomers and template in the pre-polymerization mixture is commonly singled out as the defining factor for the success of non-covalent imprinting protocol. Not surprisingly, a substantial research effort in the last two decades has been dedicated to the understanding and characterization of association processes in the pre-polymerization solution [2]. The majority of the early contributions to the field focus on the interactions between a single template molecule and one or few functional monomers. Various types of quantum mechanical, classical molecular dynamics and energy minimization methods have been employed to characterize these interactions. A number of important results emerged from these studies, including computational screening protocols to identify the most promising functional monomers for a particular template based on the strength of their interaction and degree of complementarity [3–5].

Recent experimental and simulation studies indicate that the presence of the cross-linker and solvent components cannot be ignored in the analysis of the pre-polymerization processes [6,7]. Within more sophisticated models that explicitly include these species into consideration, a picture of the pre-polymerization mixture emerges as a system with several parallel and competing association processes, including self-association of the template and functional monomer, and associations between the template and cross-linker. These processes are not independent from each other, and the final characteristics of the formed MIP, such as the binding site distribution and selectivity, are functions of all these processes.

Other processes, namely polymerization and adsorption in the imprinted materials, have received substantially less attention. Recent examples include a study by Yungerman and Srebnik, where a simplified model was applied to investigate porosity and pore size distribution in the imprinted structure as function of the template concentration and degree of polymerization [8], and an atomistic-level simulation by Henthorn and Peppas, who applied kinetic gelation technique to polymerization of 2-hydroxyethyl methacrylate (HEMA) imprinted with glucose in water solution [9]. Atomistic modeling of adsorption and molecular recognition in imprinted materials remains virtually unexplored [10].

Despite this, the performance of a MIP is assessed by its ability to recognize and rebind template molecules. This ability implies presence of specific binding sites, formed and preserved during various stages of MIP synthesis, with the structure and interaction patterns complementary to the template. In the absence of systematic studies of adsorption in model MIPs, the link between molecular recognition, the very property of MIPs that makes these materials unique and useful, and the complexation processes in the pre-polymerization solutions, remains elusive.

The objective of this and several of our earlier publications [10–13] is to establish this link by developing a model of an imprinted polymer that would satisfy the following criteria: it must capture the process of MIP formation with a sufficient degree of realism; it must generate three dimensional structures that feature a complex porous network and binding sites of various types and quality; ideally, this model should exhibit molecular recognition so the characteristics and factors affecting this phenomenon can be explored in a systematic way.

**Figure 1.**
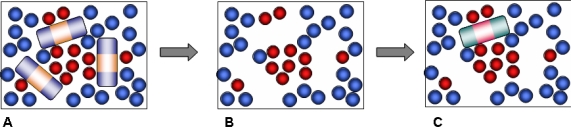
Schematic depiction of the general simulation strategy. (**A**) Pre-polymerization stage: Template species (red and blue striped cylinders) are mixed with functional monomers and other components of the mixture (blue and red particles); (**B**) Polymerization stage: molecules are frozen in their respective positions and orientations, and the template molecules are removed; (**C**) Adsorption stage: the porous matrix generated at the previous stage serves as a model molecularly imprinted polymer (MIP) in adsorption simulation, where it rebinds the template or adsorbs a close structural analogue (cyan and pink striped cylinder).

Our approach is based on a computational strategy which involves several steps, reflecting the actual experimental synthetic procedure (Figure 1). We start with a simulation of the pre-polymerization mixture, which includes template, functional monomer, cross-linker and solvent components.

This is followed by the polymerization step. As has been already discussed, in principle, advanced simulation schemes, such as those based on kinetic gelation, are available to model the process of chemical bond formation and polymerization [9]. Instead, we adopt a simpler scheme where polymerization is effectively captured by freezing the molecules of the polymerization mixture in their instant positions, orientations and conformations. In other words, various complexes and molecular arrangements corresponding to a particular configuration in the pre-polymerization mixture remain intact upon polymerization in this simplified protocol. In reality, polymerization is most likely to have a detrimental effect on these complexes, and therefore the model materials obtained within this protocol and their molecular recognition performance can be considered as a limiting, ideal case. The quenching step is followed by the template and solvent removal. The remaining structure consists of functional monomers and cross-linkers only (Figure 1B). It features a complex, three dimensional network of pores, as well as smaller cavities, complementary in their structure to the template molecules. These cavities are the imprinted binding sites and should be able to recognize and rebind the template. The molecular structure generated in this fashion serves as a model MIP material in the consequent adsorption simulation studies, aimed to assess and characterize the molecular recognition functionality of the model MIPs. At this stage all binding sites are considered to be accessible, which is not the case in real MIP structures.

A simplified treatment of the polymerization step has important theoretical implications. From the statistical mechanical point of view, adsorption in the model structures generated within the described protocol is a special case of a quenched-annealed system, where one component (MIP) is quenched, and the other component (adsorbate) is in the equilibrium, annealed state [14–19]. Thus, the statistical mechanical formalism developed to deal with the quenched-annealed systems should serve as a starting point in our understanding of model MIPs. The described protocol is general, and models of various levels of molecular detail can be constructed, depending on the type of questions they are meant to address. The first fully atomistic model of a MIP, using this protocol, was explored in our previous publication [10]. We considered a system based on methacrylic acid (MAA) and ethylene glycol dimethacrylate (EGDMA) and templated with pyridine in a chloroform solution. The model MIP structures exhibited preferential adsorption of pyridine over toluene or benzene. Strictly speaking, this selectivity cannot be considered as a purely molecular recognition effect since, in this case, pyridine would interact more strongly compared to toluene or benzene with any porous material, including a non-imprinted polymer, due to the presence of a nitrogen atom and higher polarity.

In this article we consider MAA/EGDMA systems templated with either pyrazine (PRZ) or pyrimidine (PMD). One may view the selected templates as ideal species for the simulation studies of imprinting and molecular recognition effects. They are simple and rigid, and differ only in the interaction pattern (*i.e.*, in the location of the nitrogen atoms). The goal is to verify whether atomistic models of MIPs, imprinted with these molecules, are capable of molecular recognition. This would manifest itself in pyrazine templated material being able to preferentially adsorb pyrazine over pyrimidine, and vice versa, pyrimidine templated structure should exhibit selectivity towards pyrimidine with respect to pyrazine.

## 2. Methodology

### 2.1. Systems and Molecular Force Fields

**Figure 2.**
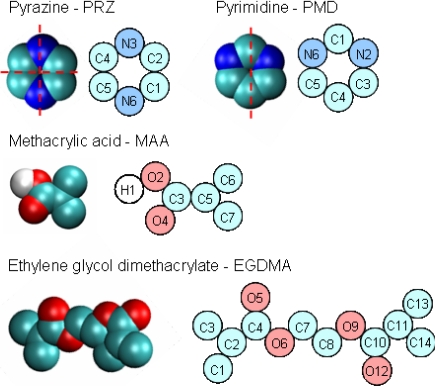
Computer visualization and schematic representation of the species involved in the atomistic simulations of model MIPs. Cyan, blue, red and white colors correspond to the carbon based united atoms (C, CH_x_), nitrogen, oxygen and explicit hydrogen atoms, respectively. Red dashed lines across the templates delineate axes of symmetry in these molecules. Interaction parameters for the atoms are summarized in Tables 2–5, according to the notation in the schematic representations of the species.

We consider methacrylic acid (MAA) as the functional monomer and ethylene glycol dimethacrylate (EGDMA) as the cross-linker, since these are some of the most commonly used components in MIP synthesis, and a significant number of well documented, reference systems are based on MAA and EGDMA [2,20]. In the previous simulation study [10], a system based on MAA and EGDMA, and templated with pyridine in chloroform solution was considered, with 1:4:20 template to functional monomer to cross-linker molar proportions to reflect a reference experimental system [20]. Unlike pyridine, pyrazine and pyrimidine have two functional groups (nitrogen atoms). Hence, to maintain the same proportion of functional monomers per functional group, twice the number of functional monomers is used in the system. In principle, a solvent such as chloroform can also be included in the system. In the previous study, the amount of solvent was varied as a way to control porosity of the final imprinted structures; it was shown that lower amounts of solvent led to higher selectivities of the model materials [10]. Here, for simplicity, an extreme case of no solvent at all is investigated. The resulting compositions of the systems considered in this article are presented in Table 1. Visualization of the species considered in this study is provided in Figure 2. Here and throughout the article we use Visual Molecular Dynamics (VMD) to produce visualizations of the systems [21].

Lennard-Jones potential $u_{\mathrm{LJ}}=4ɛ\left[{\left(\frac{\sigma }{r}\right)}^{12}-{\left(\frac{\sigma }{r}\right)}^{6}\right]$, where *ɛ*, *σ* and *r* are the well depth, collision diameter and distance between two atoms respectively, is used to describe non-polar intermolecular interactions, whereas polar interactions are described using the Coulomb potential $u_{\mathrm{COUL}}=\frac{q_{i}q_{j}}{4\pi {ɛ}_{0}r}$, where *q_i_* and *q_j_* are partial charges on atoms *i* and *j*, and *ɛ*_0_ is the electric constant. For intramolecular interactions, both PRZ and PMD are considered as rigid molecules, whereas MAA and EGDMA molecules are allowed to have bending and torsional degrees of freedom. The force field parameters for the species in this study are taken from TraPPE force field of Siepmann and co-workers [22–30]. Specifically, pyrazine and pyrimidine parameters are taken directly form TraPPE, whereas the MAA parameters are taken from TraPPE and a TraPPE-like model by Clifford and co-workers for saturated carboxylic acids [31]. This was validated by Herdes and Sarkisov by simulation of MAA vapor-liquid equilibrium data [10]. In the same work, EGDMA was modeled as two MAA molecules and a bridging ethylene glycol. Parameters for ethylene glycol were also taken from TraPPE directly. Liquid EGDMA was modeled at 1 atm and 298 K, to obtain a reasonable agreement (in terms of density) with the reference experimental data [10]. Intermolecular interaction parameters are summarized in the Tables 2–5.

### 2.2. Simulation of the Pre-Polymerization Mixture Using NPT Molecular Dynamics

The initial configurations of the systems, with the compositions specified in Table 1, are prepared by placing all the molecules in a simulation box, using a simple Monte Carlo code. The system is then equilibrated via a molecular dynamics simulation in the NPT ensemble. In these simulations, temperature is set to T = 298 K and pressure is set to P = 1 atm, to reflect typical laboratory conditions. All molecular dynamics simulations are performed using the Gromacs simulation package [32]. The simulation parameters follow those used in our previous work [10]. Equilibration is done with the time step of 0.002 ps and at least 5 × 10^6^ time steps (10 ns). Periodic boundary conditions are used for the simulation box. The LINCS algorithm is employed to constrain the molecular bonds. Berendsen coupling scheme is adopted for isotropic baro- and thermostat [33]. Particle Mesh Ewald (PME) method is used for the electrostatic calculations [34,35]. Lennard-Jones interactions are cut off at 14 Å, with long tail energy corrections applied. The systems created measure about 42 Å in size (the edge of the cubic box). Simulation for each of the two systems is repeated three times. The data for energy contributions and complex distribution is averaged over the three independent simulations and presented with the corresponding standard errors of the mean. The pre-polymerization mixtures with PRZ and PMD have densities of 1045.4 ± 5.6 g·L^−1^ and 1048.2 ± 3.9 g·L^−1^, respectively (values averaged over three simulations).

### 2.3. Simulation of Adsorption in Model MIPs

After template removal, the final configurations obtained at the previous molecular dynamics stage are used as model MIP matrices. For each MIP system three independent matrix realizations are generated. To improve statistics, adsorption is simulated on a larger supercell composed of eight replicas of each matrix realization. Monte Carlo simulations are performed in the grand canonical ensemble, where the volume and temperature of the system are fixed, as well as the chemical potential of the adsorbing species. For convenience, this chemical potential is converted to the fugacity of the adsorbing species $\frac{f}{k_{B}T}=\frac{q_{\mathrm{rot}}}{\Lambda^{3}}e^{\mu /k_{B}T}$, where *f* is fugacity, *k_B_* is the Boltzmann constant, *T* is temperature as per convention, *q_rot_* is the ideal gas rotational partition function, Λ is the de Broglie wavelength, and *μ* is the chemical potential. Final configurations obtained at a particular value of fugacity are used as initial configurations for the simulation at the next value of fugacity. As a result, a series of values for the amount adsorbed is generated as a function of increasing fugacity, which constitutes an adsorption isotherm.

Adsorption simulations are performed using the Multipurpose Simulation Code, MuSiC [36]. An energy biased grand canonical Monte Carlo (EB-GCMC) protocol is employed, as proposed by Snurr and co-workers [37], where energy maps are constructed prior to each simulation to bias insertion and deletion trials towards accessible regions of the porous space. These maps describe and store information about adsorbate-adsorbent interactions. To generate an energy map for the Coulombic term, we use a standard Ewald summation method [38]. In the generation of an energy map for the Lennard-Jones term, potentials are cut off at 10.6 Å, with no corrections applied. For the adsorbate-adsorbate Coulombic interactions we employ a variant of the Wolf pair-wise summation method [39], proposed by Fennell and Gezelter [40]. Both Coulombic and Lennard-Jones adsorbate-adsorbate interactions are cut off at 10.6 Å. The trials (insertions, deletions, rotations, translations) are given equal probability of selection. Simulation runs correspond to at least 20 × 10^6^ trials per point on the isotherm, with half of the trials being used to average properties. All the presented results correspond to simulations at T = 298 K, averaged over three independent matrix realizations and presented along with the standard error of the mean where appropriate.

## 3. Results

### 3.1. Adsorption in Model MIPs

**Figure 3.**
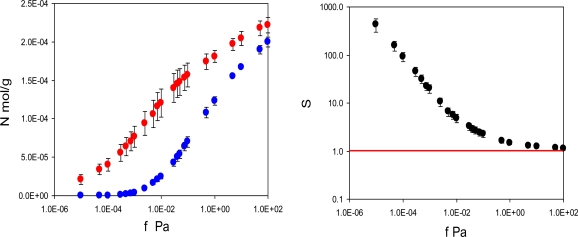
Left graph: adsorption isotherms N (mol/g) for PRZ (red circles) and PMD (blue circles) in MIP_PRZ material as a function of fugacity *f* (Pa) at 298 K. Right graph: separation factor *S (PRZ/PMD)*, defined in the text, as a function of fugacity *f* (Pa) in MIP_PRZ material at 298 K. Note the logarithmic scale for the separation factor on the right. The red line on the right corresponds to *S* = 1 (no selectivity).

In practice, performance of imprinted materials is assessed in adsorption or rebinding experiments. Hence, this is the starting point of our analysis. We have two model materials: MIP_PRZ, imprinted with PRZ, and MIP_PMD, imprinted with PMD. For each material we simulate two adsorption processes. For MIP_PRZ we consider rebinding of the template (PRZ) and adsorption of a close structural analogue (PMD). Similarly, for MIP_PMD we consider rebinding of the template (PMD) and adsorption of a close structural analogue (PRZ). For MIP_PRZ, the adsorption isotherms at 298 K for two species are shown in Figure 3. It is clear that the template molecule (PRZ) exhibits significantly stronger adsorption compared to the analogue (PMD). A more convenient way to characterize this preferential adsorption behavior is to consider the separation factor:

(1) $$S(\mathrm{PRZ}/\mathrm{PMD})=\frac{K_{\mathrm{PRZ}}}{K_{\mathrm{PMD}}}$$

(2) $$K_{i}=\frac{N_{i}(f_{i})}{B_{i}(f_{i})}$$

where *K_i_* is the partition coefficient for species *i*, relating the equilibrium loading *N_i_* at fugacity *f_i_* to the equilibrium bulk concentration *B_i_* of adsorbing species at the same fugacity. With the saturated vapor pressures for PRZ and PMD being 2490 Pa and 2780 Pa at T = 298 K respectively [41] and the upper fugacity limit on the simulated adsorption isotherms equal to 100 Pa, it is reasonable to treat the coexisting bulk phase as ideal gas. In this case, for a particular value of fugacity f* (equal for both components) the separation factor becomes

(3) $$S(\mathrm{PRZ}/\mathrm{PMD})=\frac{N_{\mathrm{PRZ}}(f*)}{N_{\mathrm{PMD}}(f*)}$$

which is simply a ratio of two adsorbed densities corresponding to a specific point on the adsorption isotherm. This data is presented in Figure 3 on the right, showing that the separation factor in this model system can exceed 400. The first three points in this graph have a substantial degree of uncertainty associated with them due to very low loadings of the analogue in MIP_PRZ. We will return to the possibility for the separation factors being 10^2^ in order of magnitude in the Discussion section.

**Figure 4.**
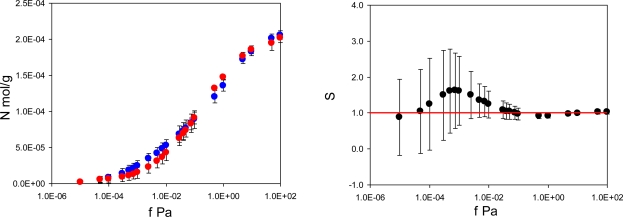
Left graph: adsorption isotherms N (mol/g) for PRZ (red circles) and PMD (blue circles) in MIP_PMD material as a function of fugacity *f* (Pa) at 298 K. Right graph: separation factor *S (PMD/PRZ)*, defined in the text, as a function of fugacity *f* (Pa) in MIP_PMD material at 298 K. The red line on the right corresponds to *S* = 1 (no selectivity).

Figure 4 summarizes adsorption isotherms of the template (PMD) and the analogue (PRZ) in the PMD imprinted material, MIP_PMD, together with the same data in the form of the separation factor $\left(S(\mathrm{PMD}/\mathrm{PRZ})=\frac{N_{\mathrm{PMD}}(f*)}{N_{\mathrm{PRZ}}(f*)}\right)$. Remarkably, unlike MIP_PRZ, MIP_PMD shows no molecular recognition with respect to its template.

**Figure 5.**
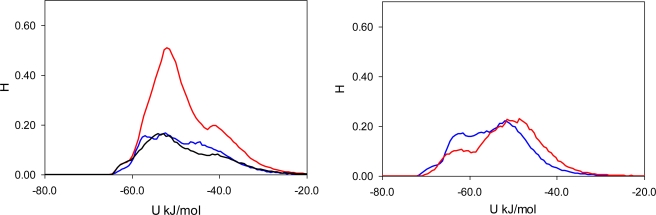
Lennard-Jones energy distribution histogram for PRZ (red line) and PMD (blue line) molecules at *f* = 0.1 Pa (intermediate loading) in MIP_PRZ (left graph) and MIP_PMD (right graph). The black line on the left corresponds to PRZ in MIP_PRZ at *f* = 5 × 10^−4^ Pa (low loading).

**Figure 6.**
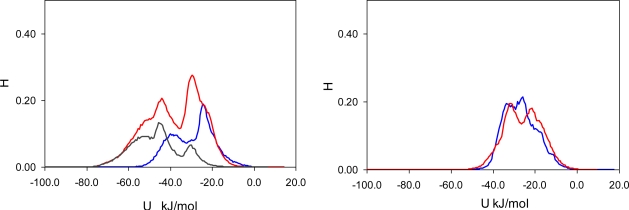
Coulombic energy distribution histogram for PRZ (red line) and PMD (blue line) molecules at *f* = 0.1 Pa (intermediate loading) in MIP_PRZ (left graph) and MIP_PMD (right graph). The black line on the left corresponds to PRZ in MIP_PRZ material at *f* = 5 × 10^−4^ Pa (low loading).

**Figure 7.**
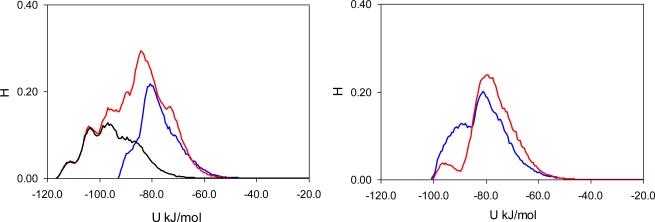
Total potential (Lennard-Jones + Coulombic) energy distribution histogram for PRZ (red line) and PMD (blue line) molecules at *f* = 0.1 Pa (intermediate loading) in MIP_PRZ (left graph) and MIP_PMD (right graph). The black line on the left corresponds to PRZ in MIP_PRZ at *f* = 5 × 10^−4^ Pa (low loading).

To elucidate the nature of molecular recognition in MIP_PRZ (and absence of this effect in PRZ_PMD), it is useful to characterize interactions of an adsorbing molecule with the porous environment. For this we calculate the energy histograms for the two model materials. An energy histogram shows the distribution of adsorbed molecules over different interaction energies, with the integral of this histogram corresponding to the adsorbate-adsorbent energy of interaction at a given loading. Initially, we consider a particular value of fugacity (*f* = 0.1 Pa), corresponding to an intermediate loading where both materials exhibit essentially no recognition (*S(PRZ/PMD)* = 1.23 and *S(PMD/PRZ)* = 0.99). Energy histograms at this value of fugacity are presented separately for the Lennard-Jones contribution (Figure 5), Coulombic contribution (Figure 6) and total (Lennard-Jones + Coulombic) energy (Figure 7). In these figures, graphs on the left correspond to MIP_PRZ material, whereas graphs on the right describe energy distributions in MIP_PMD material. The red lines correspond to PRZ as the adsorbate and the blue lines correspond to the adsorbing PMD.

Let us first concentrate on the Lennard-Jones term. For the moment we are not concerned with the overall height of the energy histogram as this property is simply proportional to the loading. The breadth of the distribution and the location of the peaks are, however, important. The Lennard-Jones energy distributions shown in Figure 5 demonstrate that, in each material, both template and analogue molecules explore the same range of energies. This suggests that the difference in behavior of PRZ and PMD in MIP_PRZ must be associated with the Coulombic contribution. Indeed, in case of MIP_PMD, Coulombic energy distributions for PRZ and PMD are very close to each other, exploring energies between −50 kJ/mol and 0 kJ/mol (Figure 6, graph on the right). In contrast, in MIP_PRZ, the energy distributions for both PRZ and PMD have a distinct bimodal shape. This shape is significantly more pronounced for PRZ and the whole distribution for this adsorbate explores much lower values of energy compared to PMD. The first peak in the PRZ distribution is centered around −44 kJ/mol. This peak is associated with adsorption in specific binding sites. The second peak for PRZ in MIP_PRZ (Figure 6, graph on the left) is around −29 kJ/mol and corresponds to filling the remaining, less specific pores.

Here, it is important however to emphasize, that the total energy distribution cannot be obtained simply as a linear combination of the Lennard-Jones and Coulombic histograms. A particular configuration, corresponding to a strong interaction on the Coulombic histogram, may at the same time be located on the unfavorable spectrum of the Lennard-Jones energies and vice versa. Thus, it is important to explore the total potential energy distributions, shown in Figure 7. In the MIP_PMD, material energy distributions for PMD (the template) and PRZ (the analogue) are very similar; they explore the same range of values. A different picture is observed in MIP_PRZ material (the left graph). Although both distributions have peaks close to −80 kJ/mol, the distribution for PRZ (template in this case) is significantly broader and skewed towards much lower energies compared to the PMD distribution. This distribution with the lower limit reaching −117 kJ/mol reflects adsorption of PRZ in very specific binding sites.

Fugacity of *f* = 0.1 Pa corresponds to an intermediate loading and in this regime, the energy histograms reflect filling both specific and non-specific binding sites. The specific binding sites should be occupied first on the adsorption isotherm and to describe this process, we add black lines to the left-hand graphs in Figure 5, 6 and 7 corresponding to the energy distributions for PRZ adsorbing in MIP_PRZ at a low value of fugacity *f* = 5 × 10^−4^ Pa. These distributions now reflect the range of interaction energies at very low loadings where primarily specific binding sites are occupied. In this regime, the total energy of interaction is shifted to much lower values and it is the Coulombic contribution that is responsible for this shift.

To ascertain the role of the Coulombic interactions in molecular recognition, we perform the following test. We simulate adsorption of PRZ and PMD in MIP_PRZ material with only Lennard-Jones interactions included. Indeed, this test shows that in the absence of Coulombic interactions, MIP_PRZ exhibits no molecular recognition towards PRZ (if anything, it actually shows a slight preference towards PMD).

So far, we established that MIP_PRZ features a population of binding sites capable of molecular recognition. Analysis of the energy distributions suggests that polar associations must be responsible for this behavior. What is the character of these associations and what is a particular arrangement of atoms within the binding site that allows these associations to form? To answer these questions, we need a systematic way to classify and detect various types of associations. This will be the objective of the next section.

### 3.2. Classification and Characterization of Dominant Molecular Associations

**Figure 8.**
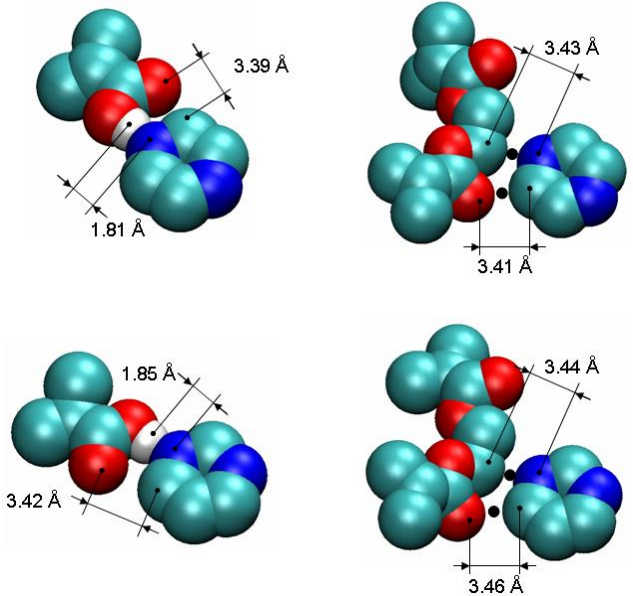
Computer visualizations of the lowest potential energy complexes obtained from the simulated annealing. Complexes involving one template and one methacrylic acid (MAA) molecule are shown on the left. Complexes involving one template and one ethylene glycol dimethacrylate (EGDMA) molecule are shown on the right. Complexes with PRZ are shown on the top, and complexes with PMD are shown in the bottom pictures. Hydrogen bonds in the complexes with MAA are self-evident. Black dots in the pictures for the complexes involving EGDMA indicate specific interactions between the two molecules responsible for the formation of the complex. These interactions are discussed in the text.

**Figure 9.**
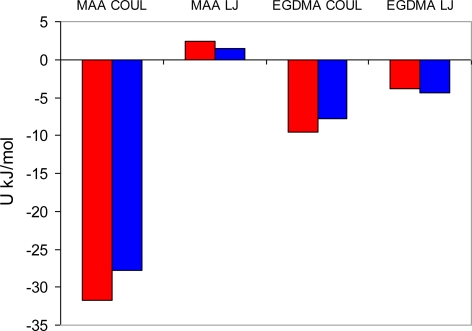
Coulombic (COUL) and Lennard-Jones (LJ) potential energy contributions observed in the lowest energy complexes, shown in Figure 8, involving one template molecule and either one MAA or one EGDMA molecule. Red bars correspond to the complexes with PRZ, and blue bars correspond to the complexes with PMD, respectively.

To detect and characterize associations within a binding site, we need an unambiguous definition of an association. To develop this definition, we consider individual complexes that form between a template molecule and other components of the pre-polymerization mixture (MAA and EGDMA in our case). For this, we perform a simulated annealing of a system consisting of single template molecule and a single molecule of either MAA or EGDMA. In simulated annealing, the system is cooled down within a molecular dynamics protocol, leading to formation of a complex corresponding to an energy minimum. To ensure that the system is not trapped in some local energy minimum, the system goes through several cycles of heating and cooling and molecular configurations at the end of each cooling phase are compared to one another. The most stable PRZ-MAA (−29.4 kJ/mol), PMD-MAA (−26.3 kJ/mol), PRZ-EGDMA (−13.5 kJ/mol) and PMD-EGDMA (−12.2 kJ/mol) complexes corresponding to the lowest potential energy of interaction (given above in the brackets for each complex) are shown in Figure 8, along with selected characteristic distances between atoms. Figure 9 compares various energy terms associated with these complexes. As expected, MAA molecule forms a hydrogen bond with one of the nitrogen atoms of the aromatic ring. Formation of this bond is driven by Coulombic interactions. Close proximity of atoms within this bond also leads to a small overlap of atoms and as a result slightly positive Lennard-Jones contribution to the total energy. An additional contribution to the total energy of the complex comes from O4 atom of the MAA molecule interacting with one of the carbons (C1, C2, C4, C5) of the PRZ molecule (with oxygen being negatively charged and carbons being positively charged). Essentially, this is also a hydrogen bond-like interaction, captured within the united atom representation (in the absence of explicit hydrogens) simply through the partial charges on the appropriate molecular fragments. Interestingly, in the PMD-MAA complex, it is not a C1 atom (charged 0.66 e) that forms an interaction with the oxygen of MAA, but either C3 or C5 (depending on what nitrogen engages in the hydrogen bond). The total interaction is stronger in the PRZ-MAA complex, compared to PMD-MAA, by about 3 kJ/mol.

Within the employed model, complexes with EGDMA, for both PRZ and PMD are governed by two primary interactions. The first interaction involves a negatively charged nitrogen atom of the template molecule and positively charged C7 or C8 atom of EGDMA (at about 3.4 Å in the lowest energy conformation), whereas the second interaction links the positively charged carbon atom of the aromatic ring (the one that is next to the engaged nitrogen atom) with the negatively charged O5 or O12 oxygen atom of the EGDMA (also at about 3.4 Å in the lowest energy conformation). In Figure 8 we mark the location of these interactions with black dots. Again, these are essentially two hydrogen bonds-like interactions, captured within the united atom representation (in the absence of explicit hydrogens) simply through the partial charges on the appropriate molecular fragments.

Again, for PMD it is C3 or C5 that is engaged in this interaction, rather than more positively charged C1. Complexes with EGDMA are much weaker than complexes with MAA, and PRZ-EGDMA complex is more energetically favorable than PMD-EGDMA.

These are the elementary associations between one template molecule and one molecule of another species. Various combinations of these associations lead to complexes of different types in solution and different binding sites in the imprinted material. We focus on several principal scenarios of complexation, defined here as follows: a complex of TM type corresponds to a template (T) molecule associated with exactly one functional monomer (M) through the hydrogen bond, with no other associations; a complex of TM2 type describes states of the template molecule where both functional groups are associated with the functional monomers; TX corresponds to the case where the template molecule is associated with exactly one cross-linker (X), and has no other associations; TX2 corresponds to a complex where template molecule is engaged with two cross-linkers, and the links are formed with both functional groups of the template; finally, TMX type corresponds to the template molecule which has one functional group associated with a functional monomer and the other group with a cross-linker. Within this definition, the total number of TM + TM2 + TX + TX2 + TMX complexes cannot exceed one per molecule, since each template molecule has two functional groups.

To detect associations of different types (between T and M and between T and X), as criteria we use characteristic interactions between atoms of two associating molecules, suggested by the simulated annealing studies. For example, a single PRZ-MAA complex is detected if a nitrogen atom of the template is within 2.5 Å from the hydrogen atom of MAA *and* a carbon atom next the nitrogen atom in the template is within 4 Å from O4 atom of MAA (this classifies as a TM complex). Similarly, an association between the template and EGDMA is detected and counted if the nitrogen atom of the template molecule is within 4 Å from C7 or C8 atom of EGDMA *and* a carbon atom of the aromatic ring of the template (the one that is next to the engaged nitrogen atom as shown in Figure 8) is within 4 Å from O5 or O12 oxygen atom of the EGDMA (this complex belongs to the TX category). If, for a single template molecule, both associations with MAA and EGDMA are detected it is recognized as a TMX complex or binding site. In this definition, the characteristic distances of 2.5 Å and 4 Å correspond to the first minimum of the respective atom-atom pair distribution functions (these functions for selected cases are summarized in the Supplemental Information file).

At this stage we do not consider complexes of geometries other than those depicted in Figure 8 (in other words, complexes based on other atom-atom interactions and distances), or higher order complexes, where more than stoichiometric number of functional monomers and cross-linkers is associated with each template, or complexes involving more than one template molecule, although we recognize the potential importance of some cooperative effects.

### 3.3. Analysis of Binding Sites and the Nature of Molecular Recognition in Model MIPs

**Figure 10.**
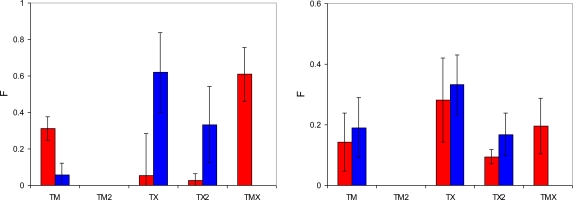
Number of associations of a particular type per molecule of template adsorbed in a model MIP structure. Data on the left corresponds to *f* = 1 × 10^−4^ Pa point on the adsorption isotherm (low loading regime). Data on the right corresponds to *f* = 10 Pa point on the adsorption isotherm (high loading regime). Classification of the complex types is provided in the text. Red bars correspond to PRZ adsorbed in MIP_PRZ, and blue bars correspond to PMD adsorbed in MIP_PMD, respectively, at 298 K. Note the change in the scale of the y-axis between the two graphs.

In this section, the classification of complexes and the criteria to detect them developed in the previous section is applied to the adsorption processes in MIP_PRZ and MIP_PMD. We consider the number of complexes of different type per molecule of adsorbate at two values of fugacity, 1 × 10^−4^ Pa, corresponding to the low loading regime, and 10 Pa, corresponding to the high loading regime. These values of fugacities are somewhat different from those used in the energy distribution analysis, and are selected to further emphasize particular features of the binding site populations. The results are summarized in Figure 10.

The most specific binding sites should emerge from molecular complexes, where two functional monomers (MAA) bind to two functional groups of the template. At least, this would be the intended behavior, and it is important to investigate whether and to what extent these associations take place. Interestingly, TM2 associations (that require formation of two hydrogen bonds with both functional groups of the template) are not observed in adsorption simulations (at least within the model system of this size). Thus, these types of sites cannot be responsible for the molecular recognition observed. TX2 is observed in a significant amount at low fugacity in MIP_PMD, and in both MIP_PMD and MIP_PRZ at high fugacity. As MIP_PMD is not a selective material, TX2 complexes should also be excluded from further consideration. One notable difference in the behavior of the two materials at low fugacity is a substantial number of TMX associations formed by PRZ in MIP_PRZ material and not observed for PMD in MIP_PMD. In fact, presence of TMX sites at both low and high loading regimes in MIP_PRZ and complete absence of these sites in MIP_PMD is of a principle importance in our proposed explanation for the molecular recognition in MIP_PRZ.

**Figure 11.**
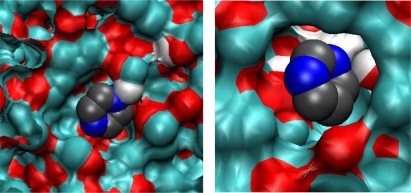
Computer visualization of a single PRZ molecule (on the left) and a single PMD molecule (on the right) in MIP_PRZ structure.

In these sites, one nitrogen atom of the binding molecule forms a hydrogen bond with an MAA molecule and the other nitrogen atom is engaged in the association with an EGDMA molecule. This type of association requires a specific arrangement of binding groups, thus making TMX type sites capable of molecular recognition. To further re-enforce this hypothesis, we perform similar binding site analysis for the PMD adsorbing in MIP_PRZ and confirm that no associations of TMX type take place. In the association of one PRZ molecule with one MAA molecule and one EGDMA molecule, Coulombic interactions should be equal to about −42 kJ/mol, according to the analysis presented in Section 3.2. This is consistent with the first peak in the energy distribution observed at a low loading (Figure 6, graph on the left), with some additional contributions coming from other molecules. Other complexes (such as TM2 and TX2) are not consistent with this value of the Coulombic interaction. To conclusively resolve this issue we examine few configurations, obtained at low fugacity, involving a single adsorbate molecule. Computer visualizations of one PRZ molecule in MIP_PRZ and one PMD molecule also in MIP_PRZ are provided in Figure 11, on the left and on the right respectively. We confirm that the PRZ molecule is indeed located in the TMX type binding site. This particular configuration is characterized by the following interactions: the Lennard-Jones term is equal to −51.59 kJ/mol, the Coulombic term is equal to −51.04 kJ/mol, with the total potential energy of interaction equal to −102.63 kJ/mol. This can be compared to a single molecule PMD configuration on the right, with the Lennard-Jones, Coulombic and total interaction energy equal to −53.07 kJ/mol, −34.05 kJ/mol and −87.12 kJ/mol, respectively.

Thus, molecular recognition in MIP_PRZ results from the presence of TMX type of binding sites. The question remains why these complexes (and consequently binding sites) form in the pre-polymerization mixture with PRZ and do not form in the pre-polymerization mixture with PMD. We address this issue in the next section.

### 3.4. Analysis of the Pre-Polymerization Mixtures

**Figure 12.**
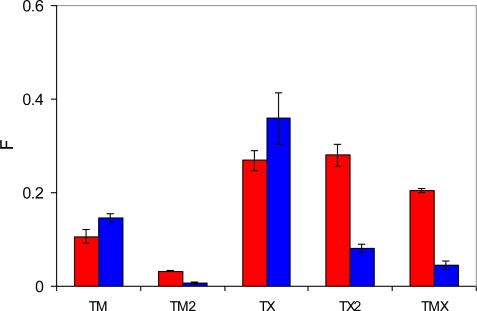
Number of associations of a particular type per molecule of template in the pre-polymerization mixture. Classification of the complex types is provided in the text. Red bars correspond to the mixture with PRZ, and blue bars to the mixture with PMD, respectively.

In this section we focus on the behavior of two pre-polymerization mixtures with the composition summarized in Table 1. The results of this analysis are presented in Figure 12, which shows the number of complexes of a particular type per molecule of the template. The first important observation is that complexes with two functional monomers (TM2) rarely form, whereas quite a substantial number of complexes with the cross-linker (TX) are formed by both PRZ and PMD. This can be rationalized using simple law of mass action arguments: although complexes with the cross-linker are energetically weaker, the EGDMA molecules are present in substantially larger numbers compared to MAA, thus shifting the equilibrium towards TX complexes. The presence of an appreciable amount of TX2 complexes can be justified on similar grounds. Another contribution to these trends comes from a strong propensity of MAA to form dimers, also observed in the previous study [10].

This figure also shows that PRZ has a much higher propensity to form TX2 and TMX complexes. Here we offer a hypothesis that implies two contributions to this trend. The first, enthalpic contribution is associated with stronger interactions between PRZ and other species, as seen from the simulated annealing studies. The second contribution is entropic in nature. Binding to either MAA or EGDMA has important consequences for the orientational degrees of freedom for PRZ and PMD. A complex involving PRZ (with one molecule of either MAA or EGDMA) can form through one of two nitrogens of PRZ, and in each case there are two equivalent possible orientations of the molecule (total of *W_PRZ_* = 4 possible configurations). For example, if the hydrogen bond is formed through N3 of PRZ, either C2 or C4 can interact with O4 of MAA, leading to the complexes of the same energy. Similarly, in the case of PMD, either nitrogen can be involved in the complex. However, upon complex formation only one unique orientation is possible for the PMD molecule (thus leading to the total of *W_PMD_* = 2 possible configurations). For example, if N2 of PMD is engaged in the hydrogen bond, it must be C3 of PMD interacting with O4 of MAA. As a result, upon complexation with either MAA or EGDMA, PMD experiences higher entropy loss compared to PRZ. The difference in the free energy of complexation between PRZ and PMD coming from this entropic contribution (or in other words the energetic advantage of PRZ over PMD), can be roughly estimated as −RTln(*W_PRZ_*/*W_PMD_*) = −1.7 kJ/mol at 298 K. Additional effects can further arise from higher crowding of molecules (or higher steric restrictions) required to form two associations with PMD, where the functional groups are slightly closer to each other than in PRZ.

We have already discussed that most likely TX2 complexes are not responsible for molecular recognition. The affinity of this complex is too weak to be consistent with the observed energy distributions. At the same time, TMX complexes, which we believe are capable of molecular recognition form in appreciable amounts in the system with PRZ. In the system with PMD, the probability to form TMX is about five times lower than that in the system with PRZ. This provides a plausible explanation why TMX sites are rarely observed in MIP_PMD and why this material is not selective. We also note here that the distribution shown in Figure 12 is similar to the one in Figure 10 on the right. This is not surprising as the graph on the right in Figure 10 considers high loading case, with the total (adsorbate + adsorbent) density of the system similar to the liquid density of the pre-polymerization mixture. One notable difference between the graph on the right in Figure 10 and Figure 12 is the shift towards TMX sites at the expense of TM2 and TX2 sites for PRZ adsorbed in MIP_PRZ.

**Figure 13.**
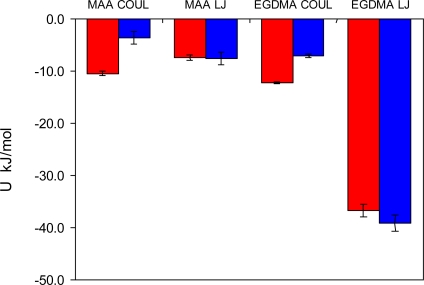
Coulombic (COUL) and Lennard-Jones (LJ) potential energy contributions per molecule of template observed in the pre-polymerization mixtures at 298 K. Red bars correspond to the system with PRZ, and blue bars correspond to the system with PMD, respectively.

The difference in the extent of complexation in the MIP_PRZ and MIP_PMD pre-polymerization mixtures can alternatively be explored through the analysis of various energy terms in the system. Figure 13 summarizes Coulombic and Lennard-Jones energy contributions observed on average between a single molecule of the template and two other components of the mixture for the two systems of interest. This distribution is clearly very different from that in Figure 9, since it describes processes in solution. The Lennard-Jones terms corresponding to either interaction with MAA or EGDMA, are similar for both templates and are governed mostly by the overall density of the system, rather than by specific interactions. Much higher concentration of EGDMA, compared to MAA, makes the Lennard-Jones term associated with EGDMA the most significant contribution to the total potential energy of interaction between a template molecule and the rest of the system. Some signature of more specific complexation can be seen in the behavior of the Coulombic terms, with PRZ exhibiting stronger interaction with both MAA and EGDMA, compared to PMD. This is a result of a higher degree of complexation in the system with PRZ and stronger interactions within complexes, involving PRZ.

## 4. Discussion

In this article we presented an atomistic model of molecularly imprinted polymers, based on mimicking the actual process of their formation. Two systems are considered, a mixture of MAA and EGDMA imprinted with pyrazine and the same mixture imprinted with pyrimidine. The performance of the two resulting materials has been assessed in a series of adsorption studies.

Model polymer imprinted with pyrazine showed a tremendous selectivity towards pyrazine with respect to pyrimidine, with selectivity reaching values of 40–50 in the low loading regime and even higher values (>400) at yet lower values of fugacity. On the other hand, a model polymer imprinted with pyrimidine shows no selectivity towards pyrimidine over pyrazine.

To rationalize this behavior, we first examine if the selectivity values of 10^2^ in order are physically meaningful. We are interested in the most specific binding sites corresponding to adsorption at low values of fugacity. The partition coefficient *K_i_* for species *i* can be related to the free energy of binding Δ*G_i_* through:

(4) $$\Delta G_{i}=-\mathrm{RT}\;\text{ln}\;K_{i}$$

This can be introduced in the expression for the separation factor:

(5) $$S(\mathrm{PRZ}/\mathrm{PMD})=\frac{e^{\frac{-\Delta G_{\mathrm{PRZ}}}{\mathrm{RT}}}}{e^{{}^{\frac{-\Delta G_{\mathrm{PMD}}}{\mathrm{RT}}}}}$$

A common treatment of the free energy of binding Δ*G_i_* would split this property into various contributions [42,43]:

(6) $$\begin{array}{l} \Delta G=\Delta G_{\mathrm{trans}+\mathrm{rot}}+\Delta G_{\mathrm{rotors}}+\Delta G_{\mathrm{conform}}\\ \;\;\;\;\;\;\;\;\;\;\;\;\;\;\;\;\;+\Delta G_{\mathrm{polar}}+\Delta G_{\mathrm{vdW}}+\Delta G_{\mathrm{solv}}+\Delta G_{\mathrm{vib}} \end{array}$$

where the free energy changes are described by changes in the translational and rotational/orientational degrees of freedom upon binding Δ*G_trans+rot_*, restriction of internal rotors in the complex Δ*G_rotors_*, adverse conformational changes upon binding Δ*G_conform_*, polar groups contribution Δ*G_polar_*, van der Waals interactions Δ*G_vdW_*, solvation effects Δ*G_solv_*, residual soft vibrational modes Δ*G_vib_* and so on.

Most of these terms contributing to the free energy of binding will be the same, or very similar for pyrazine and pyrimidine as these species are very similar. Indeed, these are small rigid molecules and we speculate that individual contributions Δ*G_rotors_*, Δ*G_conform_*, Δ*G_vib_* associated with rotors, conformational and vibrational degrees of freedom should cancel in the expression for selectivity. Analysis of the various energy terms indicate that Δ*G_vdW_* is very similar for the two species. As we consider adsorption from the gas phase (no solvent), there are no effects associated with disolvation or hydrophobic effects. The remaining terms are Δ*G_trans+rot_* and Δ*G_polar_*. So, *S(PRZ/PMD)* can be expressed as:

(7) $$S(\mathrm{PRZ}/\mathrm{PMD})=\frac{e^{\frac{-{\left(\Delta G_{\mathrm{trans}+\mathrm{rot}}+\Delta G_{\mathrm{polar}}\right)}_{\mathrm{PRZ}}}{\mathrm{RT}}}}{e^{\frac{-{\left(\Delta G_{\mathrm{trans}+\mathrm{rot}}+\Delta G_{\mathrm{polar}}\right)}_{\mathrm{PMD}}}{\mathrm{RT}}}}$$

or

(8) $$S(PRZ/PMD)=e^{\frac{-\left(\Delta G_{trans+rot}+\Delta G_{polar}\right)}{RT}}$$

It is enough for $-\Delta \left(\Delta G_{\mathrm{trans}+\mathrm{rot}}+\Delta G_{\mathrm{polar}}\right)$ to be *ca.* 6 times larger than RT for *S(PRZ/PMD)* to be more than 400; this equates to approximately a 15 kJ/mol difference in the free energy of binding between pyrazine and pyrimidine at 298 K. From the energy analysis presented above we observe that the energy differences of this magnitude between specific and non-specific binding sites, driven by polar interactions, are not uncommon.

In mixture with other components of the system, pyrazine is able to establish an appreciable number of complexes involving an association with a MAA molecule and an association with an EGDMA molecule. Upon template removal it is these complexes that become highly selective binding sites. One would expect that the mirror process should take place with pyrimidine as the template. However, pyrimidine rarely forms any complexes which would involve both of its functional groups. This is associated with a weaker interaction in the complexes, greater loss of the orientational degree of freedom for pyrimidine and, possibly, some crowding effects caused by closer proximity of the functional groups in pyrimidine. As a result, no specific sites form in MIP_PMD and no molecular recognition is observed. It would not be possible to anticipate these effects from the analysis of the energetics and structure of complexes in vacuum only.

Other key outcomes of this investigation can be summarized as follows. The presented model of MIP materials shows that the complexation processes in pre-polymerization solution can be directly linked to and are responsible for molecular recognition functionality of the resulting imprinted structures. Within this model, an interesting scenario of molecular recognition is revealed, which involves a template molecule binding to a cavity formed by a functional monomer and a cross-linker, rather than by two or more functional monomers. Thus, it is not only functional monomer—template interaction that is important in understanding of MIP performance, but interaction of the template with other species in the system as well. Ultimately, rational design of molecularly imprinted systems must consider the association processes in pre-polymerization mixture in their full complexity.

The presented model is clearly oversimplified in several aspects. Firstly, it does not consider polymerization processes explicitly, in other words, no chemical bonds are formed between the molecules in the system. Although, a high degree of cross-linking is typically required to preserve structural integrity of binding sites in MIPs, the very process of polymerization may have an adverse effect on the complexes in solution. Secondly, the original model of Herdes and Sarkisov was developed to include chloroform as a solvent [10], and the current variant of the model considers an extreme case of no solvent at all. One of the main conclusions of this article is that the association processes in the pre-polymerization mixture are strongly influenced by how molecules pack and compete with each other for the interactions. Presence of a solvent and its properties, such as size of the solvent molecules, will, naturally, affect all these processes. Furthermore, changing the nature of the solvent from apolar to polar may change the very mechanism of interactions and associations in the mixture, and type of binding sites that result from them. To capture these processes may also require some recalibration of the model to reflect a different charge distribution on the molecules in polar medium. However, in principle, both elements (explicit polymerization and presence of solvent) can be added in the model, while remaining within the general simulation protocol depicted in Figure 1. This would allow one to assess the impact of the gradual increase in the complexity of the model on the molecular recognition behavior against this reference study.

Several other aspects fall outside of the current scope of the article. This study suggests that atom-atom pair distribution functions can be efficiently used to analyze various association processes in the pre-polymerization mixture, given some definition of association types. In principle, liquid state integral equation theories, such as the reference interaction site model (RISM) [44,45] can provide a computationally attractive route to this analysis. The key elements of the theoretical formalism in application to MIP systems have been elaborated in our previous publications, but further development is required to improve the accuracy of the theoretical predictions [11,12].

The detail of the model and of the employed force fields is sufficient to generate realistic looking adsorption isotherms. One can treat these isotherms as experimental data and apply affinity distribution analysis, such as the one based on the Langmuir-Freundlich model [46], to extract information about the binding site heterogeneity. This information can then be compared to the molecular level insights on the types of binding sites present in the structure, as shown in this article. Thus, the modeling approach developed here also offers a general framework to assess the accuracy of the existing affinity distribution characterization methods, as well as to propose new ones.

## Supplementary Information



## Figures and Tables

**Table 1.** Compositions (number of molecules of each species) of model MIP systems templated with pyrazine (MIP_PRZ) and pyrimidine (MIP_PMD).

Species	MIP_PRZ	MIP_PMD
Template	10 PRZ	10 PMD
Functional monomer	80 MAA	80 MAA
Cross-linker	200 EGDMA	200 EGDMA

**Table 2.** Lennard-Jones interaction parameters (σ, ɛ) and partial charges for the atoms of the PRZ molecule, based on the TraPPE force field (see references in the text). Atom ID indicates the type and position of an atom in the molecule as shown in Figure 2.

**n**	**Atom ID**	**σ nm**	**ɛ kJ/mol**	**Charge e**
1	C1	0.374	0.399	0.33
2	C2	0.374	0.399	0.33
3	N3	0.345	0.233	−0.66
4	C4	0.374	0.399	0.33
5	C5	0.374	0.399	0.33
6	N6	0.345	0.233	− 0.66

**Table 3.** Lennard-Jones interaction parameters (σ, ɛ) and partial charges for the atoms of the PMD molecule, based on the TraPPE force field (see references in the text). Atom ID indicates the type and position of an atom in the molecule as shown in Figure 2.

**n**	**Atom ID**	**σ nm**	**ɛ kJ/mol**	**Charge e**
1	C1	0.39	0.391	0.66
2	N2	0.345	0.233	−0.66
3	C3	0.374	0.399	0.33
4	C4	0.370	0.420	0.00
5	C5	0.374	0.399	0.33
6	N6	0.345	0.233	−0.66

**Table 4.** Lennard-Jones interaction parameters (σ, ɛ) and partial charges for the atoms of the MAA molecule (see references in the text). Atom ID indicates the type and position of an atom in the molecule as shown in Figure 2.

**n**	**Atom ID**	**σ nm**	**ɛ kJ/mol**	**Charge e**
1	H1	0	0	0.37
2	O2	0.302	0.773	−0.46
3	C3	0.390	0.341	0.42
4	O4	0.305	0.657	−0.45
5	C5	0.385	0.166	0.12
6	C6	0.368	0.707	0.00
7	C7	0.375	0.815	0.00

**Table 5.** Lennard-Jones interaction parameters (σ, ɛ) and partial charges for the atoms of the EGDMA molecule (see references in the text). Atom ID indicates the type and position of an atom in the molecule as shown in Figure 2.

**n**	**Atom ID**	**σ nm**	**ɛ kJ/mol**	**Charge e**
1	C1	0.368	0.707	0.00
2	C2	0.385	0.166	0.12
3	C3	0.375	0.815	0.00
4	C4	0.390	0.341	0.42
5	O5	0.305	0.657	−0.45
6	O6	0.302	0.773	−0.46
7	C7	0.395	0.382	0.37
8	C8	0.395	0.382	0.37
9	O9	0.302	0.773	−0.46
10	C10	0.390	0.341	0.42
11	C11	0.385	0.166	0.12
12	O12	0.305	0.657	−0.45
13	C13	0.375	0.815	0.00
14	C14	0.367	0.707	0.00

## References

[1] Nicholls IA, Andersson HS, Charlton C, Henschel H, Karlsson BCG, Karlsson JG, O’Mahony J, Rosengren AM, Rosengren KJ, Wikman S (2009). Theoretical and computational strategies for rational molecularly imprinted polymer design. Biosens. Bioelectron.

[2] Alexander C, Andersson HS, Andersson LI, Ansell RJ, Kirsch N, Nicholls IA, O’Mahony J, Whitcombe MJ (2006). Molecular imprinting science and technology: A survey of the literature for the years up to and including 2003. J. Mol. Recognit.

[3] Chianella I, Lotierzo M, Piletsky SA, Tothill IE, Chen B, Karim K, Turner APF (2002). Rational design of a polymer specific for microcystin-LR using a computational approach. Anal. Chem.

[4] Karim K, Breton F, Rouillon R, Piletska EV, Guerreiro A, Chianella I, Piletsky SA (2005). How to find effective functional monomers for effective molecularly imprinted polymers?. Adv. Drug Deliv. Rev.

[5] Chianella I, Karim K, Piletska EV, Preston C, Piletsky SA (2006). Computational design and synthesis of molecularly imprinted polymers with high binding capacity for pharmaceutical applications-model case: Adsorbent for abacavir. Anal. Chim. Acta.

[6] O’Mahony J, Karlsson BCG, Mizaikoff B, Nicholls IA (2007). Correlated theoretical, spectroscopic and X-ray crystallographic studies of a non-covalent molecularly imprinted polymerisation system. Analyst.

[7] Karlsson BCG, O’Mahony J, Karlsson JG, Bengtsson H, Eriksson LA, Nicholls IA (2009). Structure and dynamics of monomer-template complexation: An explanation for molecularly imprinted polymer recognition site heterogeneity. J. Am. Chem. Soc.

[8] Yungerman I, Srebnik S (2006). Factors contributing to binding-site imperfections in imprinted polymers. Chem. Mater.

[9] Henthorn DB, Peppas NA (2007). Molecular simulations of recognitive behavior of molecularly imprinted intelligent polymeric networks. Ind. Eng. Chem. Res.

[10] Herdes C, Sarkisov L (2009). Computer simulation of volatile organic compound adsorption in atomistic models of molecularly imprinted polymers. Langmuir.

[11] Sarkisov L, van Tassel PR (2005). Replica Ornstein-Zernike theory of adsorption in a templated porous material: Interaction site systems. J Chem Phys.

[12] Sarkisov L, van Tassel PR (2007). Integral equation theory of adsorption in templated materials: Influence of molecular attraction. J. Phys. Chem. C.

[13] Dourado EMA, Sarkisov L (2009). Emergence of molecular recognition phenomena in a simple model of imprinted porous materials. J Chem Phys.

[14] Madden WG, Glandt ED (1988). Distribution-functions for fluids in random-media. J. Stat. Phys.

[15] Fanti LA, Glandt ED, Madden WG (1990). Fluids in equilibrium with disordered porous materials—integral-equation theory. J. Chem. Phys.

[16] Given JA (1992). Liquid-state methods for random-media—Random sequential adsorption. Phys. Rev. A.

[17] Madden WG (1992). Fluid distributions in random media: Arbitrary matrices. J. Chem. Phys.

[18] Given JA, Stell GR (1994). The replica Ornstein-Zernike equations and the structure of partly quenched media. Phys. A.

[19] Sarkisov L, van Tassel PR (2008). Theories of molecular fluids confined in disordered porous materials. J. Phys.-Condens. Matter.

[20] Andersson HS, KochSchmidt AC, Ohlson S, Mosbach K (1996). Study of the nature of recognition in molecularly imprinted polymers. J. Mol. Recogn.

[21] Humphrey W, Dalke A, Schulten K (1996). VMD: Visual molecular dynamics. J. Mol. Graph.

[22] Martin MG, Siepmann JI (1999). Novel configurational-bias Monte Carlo method for branched molecules. Transferable potentials for phase equilibria. 2. United-atom description of branched alkanes. J. Phys. Chem. B.

[23] Wick CD, Martin MG, Siepmann JI (2000). Transferable potentials for phase equilibria. 4. United-atom description of linear and branched alkenes and alkylbenzenes. J. Phys. Chem. B.

[24] Chen B, Potoff JJ, Siepmann JI (2001). Monte Carlo calculations for alcohols and their mixtures with alkanes. Transferable potentials for phase equilibria. 5. United-atom description of primary, secondary, and tertiary alcohols. J. Phys. Chem. B.

[25] Rai N, Siepmann JI (2007). Transferable potentials for phase equilibria. 9. Explicit hydrogen description of benzene and five-membered and six-membered heterocyclic aromatic compounds. J. Phys. Chem. B.

[26] Chen B, Siepmann JI (1999). Transferable potentials for phase equilibria. 3. Explicit-hydrogen description of normal alkanes. J. Phys. Chem. B.

[27] Lubna N, Kamath G, Potoff JJ, Rai N, Siepmann JI (2005). Transferable potentials for phase equilibria. 8. United-atom description for thiols, sulfides, disulfides, and thiophene. J. Phys. Chem. B.

[28] Martin MG, Siepmann JI (1998). Transferable potentials for phase equilibria. 1. United-atom description of n-alkanes. J. Phys. Chem. B.

[29] Stubbs JM, Potoff JJ, Siepmann JI (2004). Transferable potentials for phase equilibria. 6. United-atom description for ethers, glycols, ketones, and aldehydes. J. Phys. Chem. B.

[30] Wick CD, Stubbs JM, Rai N, Siepmann JI (2005). Transferable potentials for phase equilibria. 7. Primary, secondary, and tertiary amines, nitroalkanes and nitrobenzene, nitriles, amides, pyridine, and pyrimidine. J. Phys. Chem. B.

[31] Clifford S, Bolton K, Ramjugernath D (2006). Monte carlo simulation of carboxylic acid phase equilibria. J. Phys. Chem. B.

[32] Lindahl E, Hess B, van der Spoel D (2001). GROMACS 3.0: A package for molecular simulation and trajectory analysis. J. Mol. Model.

[33] Berendsen HJC, Postma JPM, Vangunsteren WF, Dinola A, Haak JR (1984). Molecular-dynamics with coupling to an external bath. J. Chem. Phys.

[34] Darden T, York D, Pedersen L (1993). Particle mesh ewald—An n.log(n) method for Ewald sums in large systems. J. Chem. Phys.

[35] Essmann U, Perera L, Berkowitz ML, Darden T, Lee H, Pedersen LG (1995). A smooth particle mesh Ewald method. J. Chem. Phys.

[36] Gupta A, Chempath S, Sanborn MJ, Clark LA, Snurr RQ (2003). Object-oriented programming paradigms for molecular modeling. Mol. Simul.

[37] Snurr RQ, Bell AT, Theodorou DN (1993). Prediction of adsorption of aromatic-hydrocarbons in silicalite from grand-canonical monte-carlo simulations with biased insertions. J. Phys. Chem.

[38] Frenkel D, Smit B (2002). Understanding Molecular Simulation: From Algorithms to Applications.

[39] Wolf D, Keblinski P, Phillpot SR, Eggebrecht J (1999). Exact method for the simulation of Coulombic systems by spherically truncated, pairwise r(-1) summation. J. Chem. Phys.

[40] Fennell CJ, Gezelter JD (2006). Is the Ewald summation still necessary? Pairwise alternatives to the accepted standard for long-range electrostatics. J Chem Phys.

[41] Lipkind D, Chickos JS (2009). An examination of the vaporization enthalpies and vapor pressures of pyrazine, pyrimidine, pyridazine, and 1,3,5-triazine. Struct. Chem.

[42] Nicholls IA (1995). Thermodynamic considerations for the design of and ligand recognition by molecularly imprinted polymers. Chem. Lett.

[43] Nicholls IA (1998). Towards the rational design of molecularly imprinted polymers. J. Mol. Recogn.

[44] Chandler D, Andersen HC (1972). Optimized cluster expansions for classical fluids. II. Theory of molecular liquids. J. Chem. Phys.

[45] Cummings PT, Stell G (1982). Interaction site models for molecular fluids. Mol. Phys.

[46] Umpleby RJ, Baxter SC, Rampey AM, Rushton GT, Chen Y, Shimizu KD (2004). Characterization of the heterogeneous binding site affinity distributions in molecularly imprinted polymers. J. Chromatogr. B.

