# Supplementary material for: Molecular Recognition Effects in Atomistic Models of Imprinted Polymers
Source: Int J Mol Sci. 2011 Jul 28;12(8):4781–804. doi: 10.3390/ijms12084781 (PMC3179132; doi:10.3390/ijms12084781)
Supplement: Supplementary file 1 [file ijms-12-04781-s001.pdf]

Supplementary Information

Eduardo M.A. Dourado [1], Carmelo Herdes [2], Paul R. Van Tassel [3] and Lev Sarkisov [1,*]

[1] Institute for Materials and Processes, School of Engineering, University of Edinburgh, UK
[2] Centro de Química de Évora, Universidade de Évora, Rua Romão Romalho 59, 7000 *Évora*, Portugal
[3] Department of Chemical and Environmental Engineering, Yale University, New Haven, CT 06520–8267, USA

* Author to whom correspondence should be addressed; E-Mail: Lev.Sarkisov@ed.ac.uk; Tel.: +44-131-650-4862.

Received: 10 June 2011; in revised form: 8 July 2011 / Accepted: 25 July 2011 / Published: 28 July 2011

Abstract: In this article we present a model for molecularly imprinted polymers, which considers both complexation processes in the pre-polymerization mixture and adsorption in the imprinted structures within a single consistent framework. As a case study we investigate MAA/EGDMA polymers imprinted with pyrazine and pyrimidine. A polymer imprinted with pyrazine shows substantial selectivity towards pyrazine over pyrimidine, thus exhibiting molecular recognition, whereas the pyrimidine imprinted structure shows no preferential adsorption of the template. Binding sites responsible for the molecular recognition of pyrazine involve one MAA molecule and one EGDMA molecule, forming associations with the two functional groups of the pyrazine molecule. Presence of these specific sites in the pyrazine imprinted system and lack of the analogous sites in the pyrimidine imprinted system is directly linked to the complexation processes in the pre-polymerization solution. These processes are quite different for pyrazine and pyrimidine as a result of both enthalpic and entropic effects.

Keywords: molecular; recognition; imprinted polymer; simulation; adsorption; rebinding; Monte Carlo; dynamics

---

1. Radial Distribution Functions g(r) in the MIP_PRZ and MIP_PMD Pre-Polymerization Mixtures at 298 K

Figure S1. Radial distribution functions g(r) for: (**a**) the templates (PRZ, PMD) and methacrylic acid (MAA) in the corresponding pre-polymerization mixtures. These are molecule-molecule distribution functions, calculated using the centers of mass of the molecules; (**b**) atom H1 (in MAA) and nitrogen atoms in PRZ and PMD.

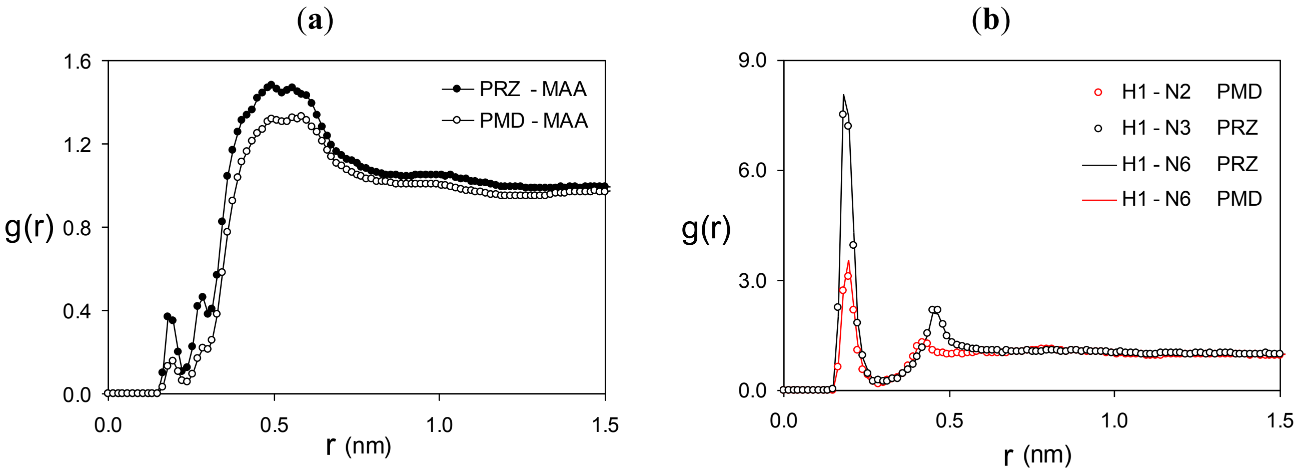

Figure S2. Radial distribution function g(r) for atom O4 (in MAA) and carbon atoms in PRZ, with C1 in blue, C2 in red, C4 in green, C5 in black.

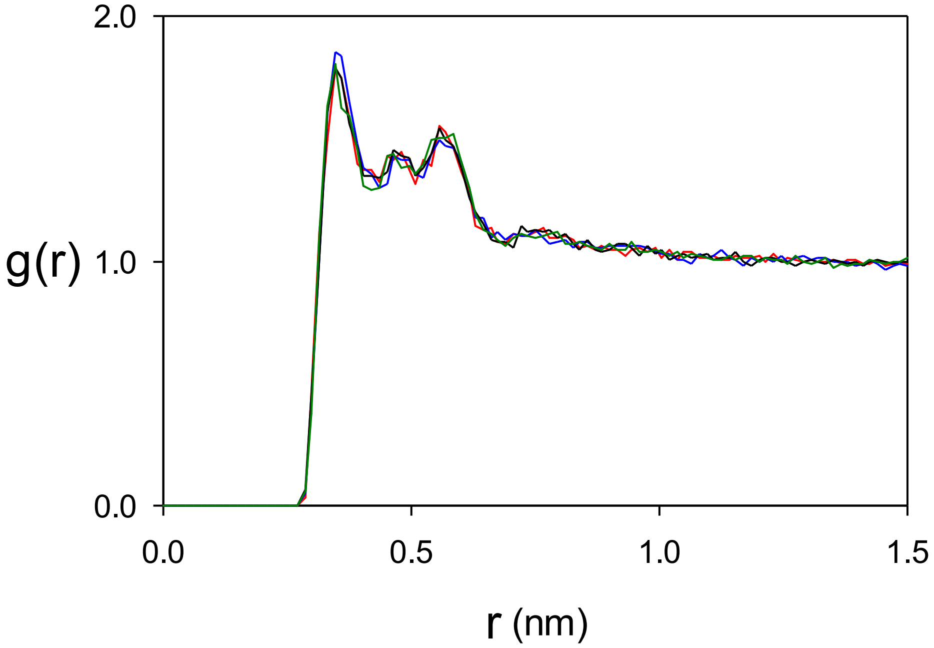

Figure S3. Radial distribution function g(r) for atom O4 (in MAA) and carbon atoms in PMD, with C1 in blue, C3 in red, C4 in green, C5 in black.

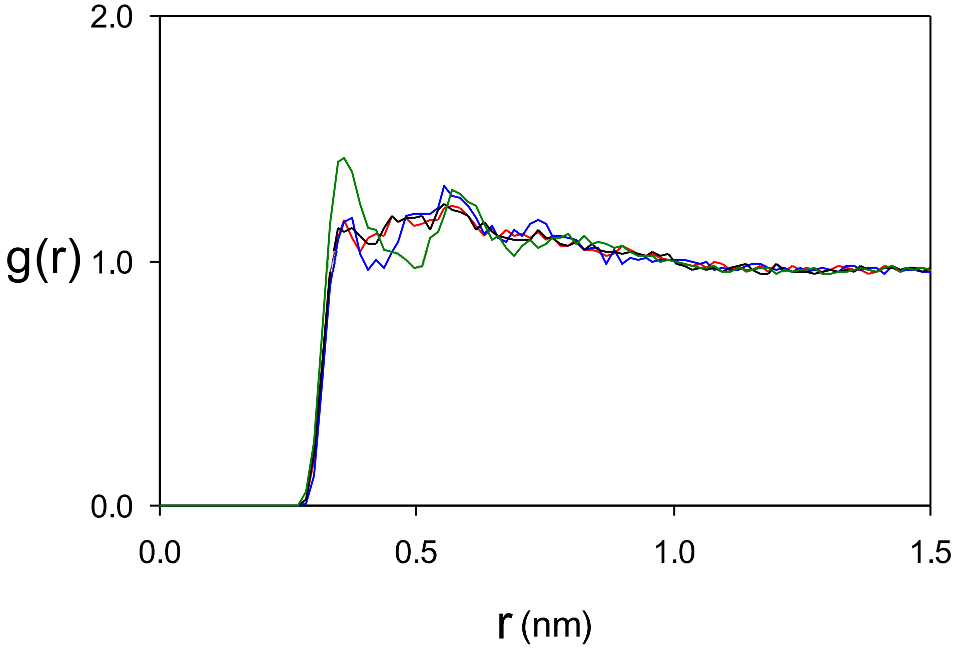

2. Computer Visualizations of Adsorption States

Figure S4. A snapshot of a typical configuration of PRZ (shown as an aromatic ring of dark blue color) adsorbing in MIP_PRZ material at 298 K.

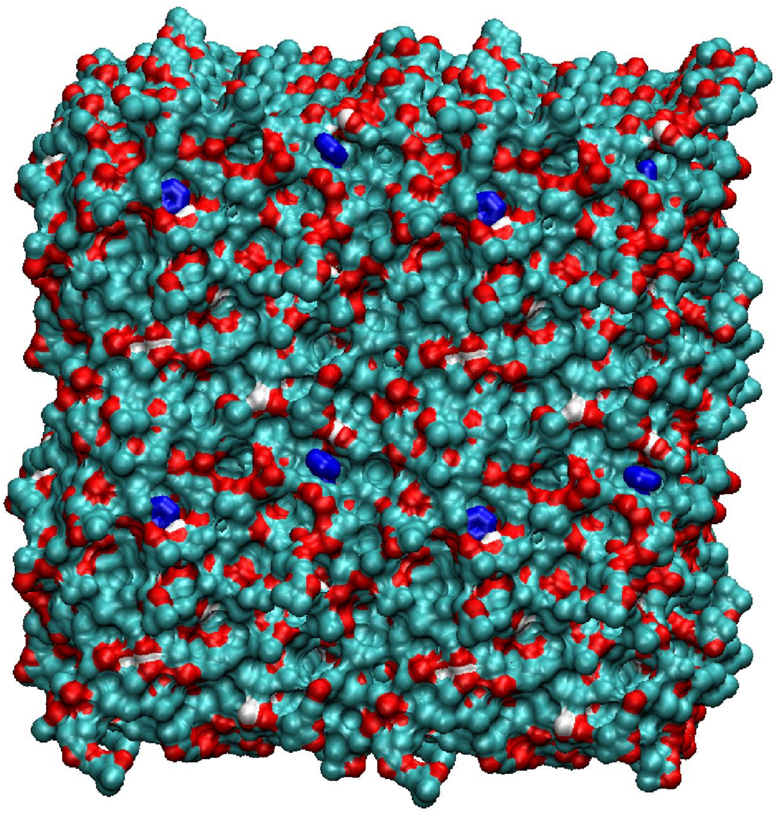

Figure S5. Computer visualizations of typical configurations of templates adsorbing in binding sites of model imprinted materials. Top row of snapshots is for PRZ in MIP_PRZ. Bottom row is for PMD in MIP_PMD and MIP_PRZ (on the right).

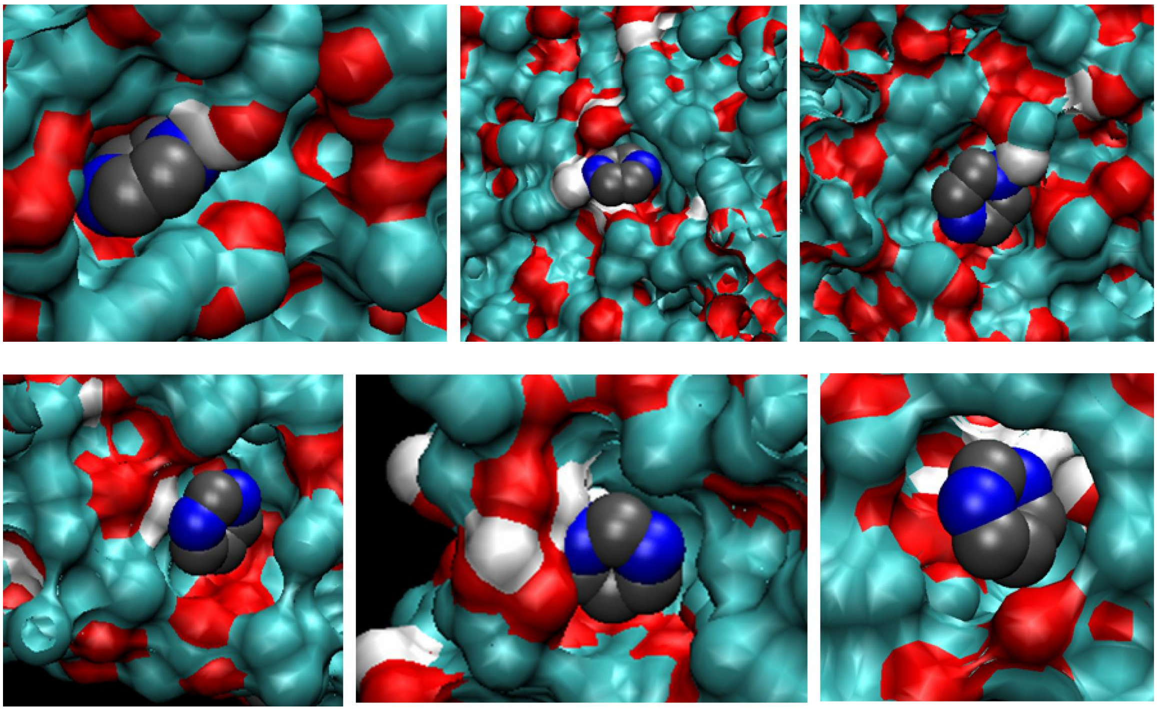

© 2011 by the authors; licensee MDPI, Basel, Switzerland. This article is an open access article distributed under the terms and conditions of the Creative Commons Attribution license (http://creativecommons.org/licenses/by/3.0/).
